# Prophylactic Effect of Echinacoside on Peripheral Neuropathy by Inhibiting Spinal Neuroinflammation and Exerting Neuroprotective Effects

**DOI:** 10.33549/physiolres.935707

**Published:** 2026-04-01

**Authors:** Xue BAI, Chenghong SUN, Kai SONG, Xiaojun ZHOU, Li WANG, Xin LI, Qian ZHOU

**Affiliations:** 1 Department of Anesthesiology, Hubei No. 3 People’s Hospital of Jianghan University, Wuhan, China; 2 Department of Pain Medicine, Hubei No. 3 People’s Hospital of Jianghan University, Wuhan, China

**Keywords:** Neuropathic pain, Paclitaxel, Echinacoside, Microglia, Astrocyte, Inflammation

## Abstract

Paclitaxel leads to peripheral neuropathy (PIPN) in approximately 50 % of cancer patients. At present, there are no effective treatment strategies, and the mechanisms of which also remain unclear. Echinacoside (ECH), a natural compound with known neuroprotective, antioxidant and anti-inflammatory properties, holds significant therapeutic potential for PIPN. However, the precise mechanism of its action remains unknown. In this study, we aimed to investigate the effectiveness of echinacoside in a mouse model of neuropathic pain induced by paclitaxel, as well as the underlying mechanisms. Administration of paclitaxel (2.0 mg/kg on four alternate days) induced acute mechanical/cold/thermal hypersensitivity in mice. Treatment with echinacoside significantly alleviated pain behavior. Furthermore, paclitaxel led to microglia and astrocyte activation and upregulation of inflammatory cytokines, including TNF-α, IL-1β, and IL-6 in the spinal cord. These neuroinflammatory responses were reversed by echinacoside treatment. Additionally, echinacoside can attenuate the loss of hind paw nerve fibers induced by paclitaxel administration. Our findings demonstrate that echinacoside reduces paclitaxel-induced neuropathic pain by attenuating microglia and astrocyte reactivity, neuroinflammatory processes in spinal cord and alleviating the loss of peripheral nerve fibers.

## Introduction

Paclitaxel is a first-line chemotherapy drug widely used to treat breast cancer. However, it often causes peripheral neuropathy, with symptoms like hand-foot syndrome appearing as early as 24–72 hours after treatment in about 50 % of patients. Among these, 30 % may experience persistent pain [1]. Currently, there is no effective treatment for paclitaxel-induced peripheral neuropathy (PIPN) [2]. Unlike other paclitaxel-related side effects (e.g., diarrhea, mucositis), PIPN is especially concerning because it is the most common reason for discontinuing or pausing chemotherapy [3]. On the basis of the antitumor mechanisms of paclitaxel, the neurotoxicity of paclitaxel may be related to their interference with the specific binding of β-microtubule proteins in neurons [4]. Along with genetic factors, molecular mechanisms such as increased neuronal excitability, neuronal oxidative stress, neuroinflammation, altered ion channel function, and glial cell activation may also be involved in the development of PIPN [5]. Yet, drugs targeting these mechanisms have failed to prevent or treat PIPN effectively. Moving forward, two key challenges remain: identifying high-risk patients and developing new strategies for PIPN prevention or treatment.

Echinacoside (ECH), a natural phenylethanoid glycoside originally isolated from Echinacea angustifolia, is also found in other plants such as Rehmannia glutinosa [6], exhibits diverse pharmacological properties, including antioxidant, anti-inflammatory, anti-infective, and anti-tumor effects [7]. Echinacoside can cross the blood-brain barrier, suggesting its potential clinical application in treating neurological disorders [8]. Accumulating preclinical evidence has established its efficacy in mitigating Parkinson’s disease, Alzheimer’s disease, cognitive dysfunction, and related neurological pathologies [6]. Nevertheless, its analgesic properties remain poorly studied. Given it’s anti-inflammatory and neuroprotective activities, echinacoside may represent a promising therapeutic candidate for paclitaxel-induced peripheral neuropathy (PIPN). The aim of this research was to study whether echinacoside could ameliorate the pain behavior in PIPN mice model and to investigate the underlying mechanisms.

## Materials and Methods

### Animals

All experimental procedures were carried out in accordance with the guidelines of the International Association for the Study of Pain and approved by the Institutional Animal Care and Use Committee of Hubei NO.3 People’s Hospital. Male C57 mice (20–25 g, 5–8 weeks,) in a temperature-controlled environment on a 12 h/12 h light/dark cycle with food and water available *ad libitum* were used.

### Treatment and experimental design

**Fig. 1:**
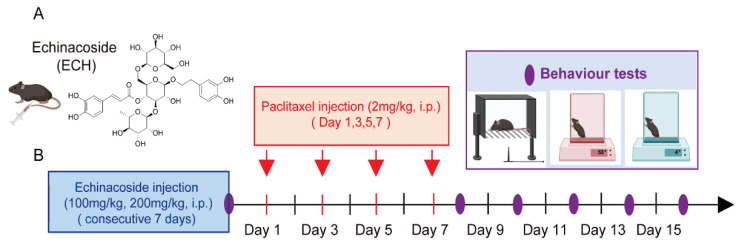
Chemical structure of Echinacoside (ECH) and protocols of this study. (**A**) Molecular formula and molecular weight of ECH are C35H46O20 and 786.7, respectively. (**B**) The schematics of the experimental design and behaviour test time points.

Neuropathic pain was induced by the intraperitoneal administration of paclitaxel in mice. Paclitaxel was dissolved in normal saline (with 4 % DMSO) before use and was injected four times a week on days 1, 3, 5, and 7 at a dose of 2 mg/kg. The ECH was dissolved in 0.9 % saline, The saline group received the same vehicle as the ECH-treated groups. The behavioral tests for pain assessment were performed at 6 time points on day 0, 8, 10, 12, 14 and 16. Echinacoside 100 or 200 mg/kg was administered once a day for 7 consecutive days before the first day of paclitaxel injection (Fig. 1).

### Mechanical allodynia (von Frey test)

For the von Frey test, each mouse was placed in an individual chamber with opaque walls on an elevated mesh rack to prevent visual or physical contact between animals during testing. The hind paw was stimulated using a series of von Frey filaments (0.16–2.56 g, Stoelting) with logarithmically increasing stiffness, applied perpendicularly to the central plantar surface. The 50 % paw withdrawal threshold was determined using the up-down method.

### Thermal/Cold hyperalgesia (hot/cold-plate test)

Thermal/Cold hyperalgesia was assessed using a hot plate analgesia meter (BIO-CHP, Bioseb, France). The animals were placed one at a time on a hot plate (53 ± 0.5 °C) or cold plate (0 ± 0.5 °C) [9]. Response latency either to jump or a hind-paw-lick was recorded. Each mouse was measured 3 times, with a 10-minutes interval between each measurement. Lifting for normal locomotion was excluded.

### Immunohistochemistry

The mice L4–L6 spinal cord and paw skins were collected and stored in 4 % paraformaldehyde solution and immobilized at 4 °C for 24 hours. Samples were then placed in 30 % sucrose for at least 24 h and included in OCT (Thermo Fisher Scientific, USA) and then sliced into 25 μm thick serial coronal sections by cryostat (Thermo Scientific, USA). Then the samples were blocked with 5 % BSA and 0.3 % Triton X-100 in PBS for 1 h at room temperature. The spinal cord sections were incubated overnight at 4 °C with primary antibodies against Iba-1 (1:500; Abcam, USA), GFAP (1:500; Abcam, USA) and paw skins incubated with PGP9.5 (1:500; Abcam, USA). After washing, the sections were then incubated with FITC-conjugated goat anti-rabbit antibody (1:800; Abcam, USA) for 1 h at room temperature. Then the nuclei were stained with DAPI. Sections were viewed under an IX51 confocal microscope (Olympus, Japan) and images were analyzed by Image-Pro Plus 6.0 software.

### Western blot analysis

L4–L6 spinal cord tissues were freshly isolated from mice and lysed in radioimmunoprecipitation assay (RIPA) (Beyotime, China). The quantity of protein of each sample was calculated using a BCA protein assay kit. Equal amounts of protein were loaded into 12 % SDS-PAGE and transferred to PVDF membranes. After being blocked with 5 % milk for 1h at room temperature, the membranes were incubated overnight at 4 °C with primary antibodies and then incubated with secondary antibodies (1:1000, Abcam, USA) for 2 h at room temperature. The primary antibodies used in Western blot include: rabbit anti-IL-1β (1:500, GB11113, Servicebio), rabbit anti-TNF-α (1:500, GB11188, Servicebio), rabbit anti-IL-6 (1:500, 12912T, CST), rabbit anti-PGP9.5 (1:500, ab108986, Abcam). Immunoblots were developed by chemiluminescent substrate and quantified using Image-J 1.80 software.

### Statistical analysis

All experimental results are given as a mean ± standard error of the mean (SEM). For multiple comparisons, one-way or two-way ANOVA was performed followed by post hoc Bonferroni’s test. *P <* 0.05 was considered to indicate a statistically significant difference. The statistical analysis was accomplished using Graph Pad Prism version 7.0 (San Diego, CA, USA).

## Results

### Echinacoside attenuated the paclitaxel-induced mechanical allodynia and thermal/cold hyperalgesia

**Fig. 2:**
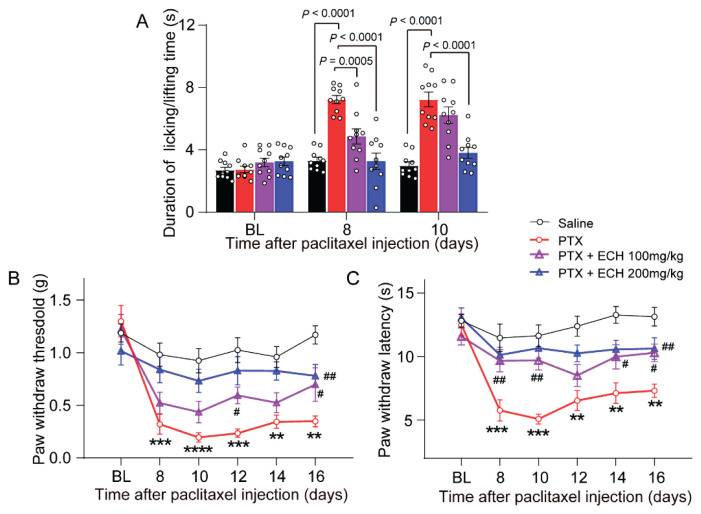
Effects of consecutive administration of ECH on noci-ceptive behavior in mice. (**A**) Effect of ECH on paw duration of lifting/licking time of mice in cold-plate test at day 0,8 and 10. (**B**) Effect of ECH on PWT of mice in Von Frey test at day 0, 8, 10, 12, 14 and 16. (**C**) Effect of ECH on PWL of mice in hot-plate test at day 0, 8, 10, 12, 14 and 16. Two-way repeated-measures ANOVA with group as the between-subjects factor and day/time as the within-subjects factor. Simple effects ANOVA was used to confirm the differences between groups at each time point. Data are expressed as mean ± SEM. ** *P* < 0.01, *** *P* < 0.001, **** *P* < 0.0001 vs. saline group; ^#^*P* < 0.05, ^##^*P*< 0.01 vs. paclitaxel group. (n = 10 mice/group)

Compared with the saline group, paclitaxel-treated mice developed reliable mechanical allodynia and thermal/cold hyperalgesia behavior as tested in von Frey and hot/cold plate tests from day 8 to 16 after the first injection of paclitaxel. This was reflected by a significant difference between groups for mechanical allodynia and paw licking latency. The most profound pain behavior appeared at day 10 (*P* < 0.001, Fig. 2A–C).

Repeated injections of echinacoside (200mg/kg), given during the PIPN induction prevented the establishment of PIPN. The paclitaxel + echinacoside (200mg/kg) treated mice showed a significant improvement of mechanical allodynia (*P* < 0.01, Fig. 2B), thermal hyperalgesia behavior (*P* < 0.01, Fig. 2C) and cold hyperalgesia behavior (*P* < 0.001, Fig. 2A) at day 10 compared with paclitaxel group. Repeated injections of echinacoside (100mg/kg) exhibited modest analgesic effects at certain time points. Echinacoside (100mg/kg) showed a significant improvement of mechanical allodynia (*P*<0.05, Fig. 2B) at day 12 and 16, thermal hyperalgesia behavior (*P* < 0.01, day 8 and 10; *P* < 0.05, day 14 and 16; Fig. 2C,) and cold hyperalgesia behavior (*P* < 0.001, day 8; Fig. 2A). These results suggest that prophylactic administration of echinacoside can alleviate the development of paclitaxel-induced pain behaviors in mice in a dose-dependent manner.

### Echinacoside decreased paclitaxel-induced spinal microglia and astrocyte activation

**Fig. 3:**
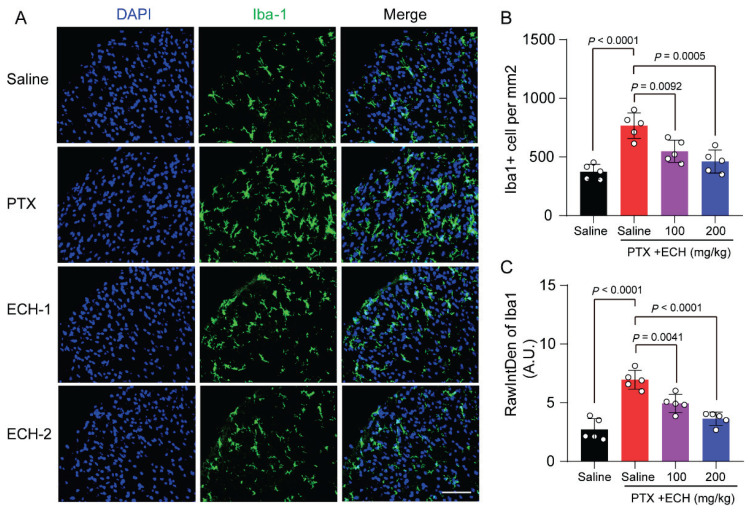
Effect of repeated ECH on the expression of microglial activation marker Iba-1 in lumbar L4-L6 spinal cord of mice. (**A**) Immunostaining for Iba-1 in spinal cord segments from mice in each group. Scale bar, 100 μm, (**B**) Quantification of the cell number of Iba1^+^ microglia of each group. (**C**) Quantification of fluorescence intensity for Iba-1 in spinal cord segments from mice in each group. one-way ANOVA, followed by Dunnett’s multiple comparisons test. P values are indicated in the Figs. Data are expressed as mean ± SEM. (n = 5 mice/group)

**Fig. 4:**
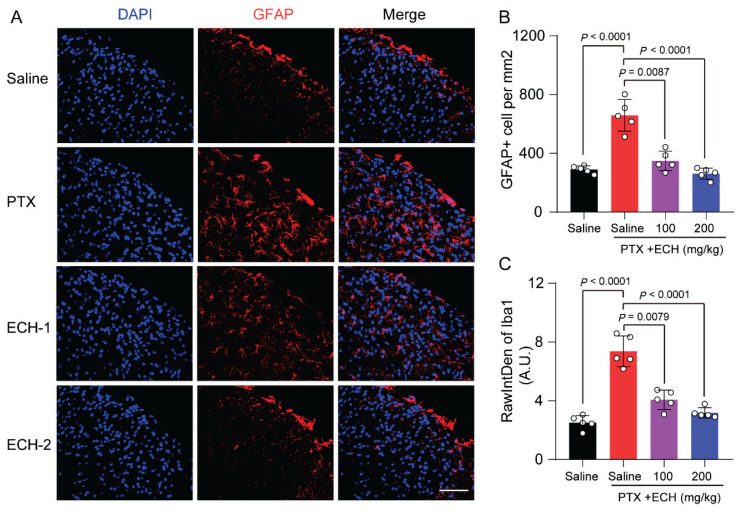
Effect of repeated ECH on the expression of astrocyte activation marker GFAP in lumbar L4–L6 spinal cord of PIPN mice. (**A**) Immunostaining for GFAP in spinal cord segments from mice in each group. Scale bar = 100 μm. (**B**) Quantification of the cell number of GFAP + astrocyte of each group. (**C**) Quantification fluorescence intensity for GFAP in spinal cord segments from mice in each group. one-way ANOVA, followed by Dunnett’s multiple comparisons test. P values are indicated in the Figs. Data are expressed as mean ± SEM. (n = 5 mice/group)

It has been reported that the activation of microglia and astrocyte plays a critical role in central sensitization in neuropathic pain. We further examined echinacoside could contribute to the formation and maintenance of neuropathic pain via the activation of microglia and astrocyte. The quantification of density and intensity of Iba1^+^ cells, suggests an increase of reactive microglia in paclitaxel treated mice (Fig. 3B–C; *P*<0.0001). Echinacoside (200mg/kg) administration significantly attenuated microglial activation (Fig. 3B–C; *P*=0.0005, *P<*0.0001, respectively). While, Echinacoside (100mg/kg) administration partially attenuated microglial activation (Fig. 3B–C; *P*=0.0092, *P*=0.0041, respectively). Likewise, Echinacoside also attenuated the activation of astrocytes in a dose-dependent manner (Fig. 4A–C).

### Repeated injections of echinacoside reduced paclitaxel-induced enhanced formation of pro-inflammatory cytokines in spinal cord

**Fig. 5:**
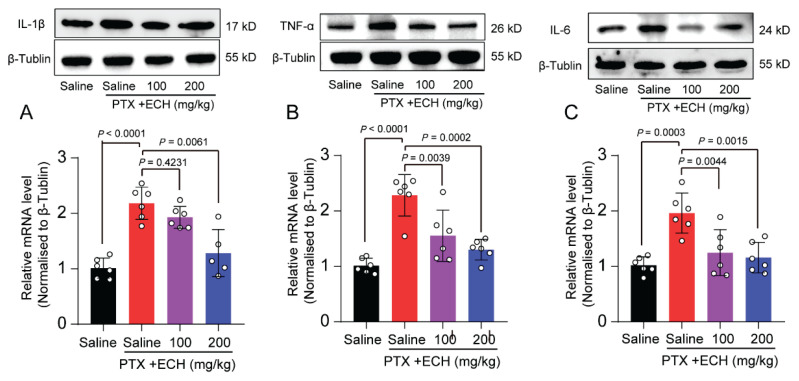
Effect of repeated ECH on the levels of proinflammatory cytokine in lumbar L5/L6 spinal cord of mice. (**A**) Western blot and quantification of IL-1β of each group mice in SDH. (**B**) Western blot and quantification of TNF-α of each group mice in SDH. (**C**) Western blot and quantification of IL-6 of each group mice in SDH. one-way ANOVA, followed by Dunnett’s multiple comparisons test. P values are indicated in the Figs. Data are expressed as mean ± SEM. (n = 6 mice/group)

We used Western Blot method to detect the relative expression of IL1β, TNF-α, IL-6 and in the spinal cord of each group of mice. The results indicated that the expression of IL-1β, TNF-α, IL-6 in spinal cord were significantly up-regulated in paclitaxel treated mice compared with saline group (Fig. 5A–C; *P*<0.0001, *P*<0.0001, *P*=0.0003, respectively). Echinacoside (200mg/kg) prophylactic administration significantly prevented the paclitaxel-induced upregulation of inflammatory cytokines. (Fig. 5A–C; *P*=0.0061, *P*=0.0002, *P*=0.0015, respectively).

### Repeated injections of echinacoside reduced paclitaxel-induced loss of nerve fibers in mice hind paw

Intraepidermal nerve fibers (IENF) are formed by nerve fibers ascending from the dermal nerve plexus, crossing the dermal-epidermal junction. In the early stages of peripheral nerve damage, IENF density decreases, making it the gold standard for evaluating small fiber neuropathy. Studies have shown that chemotherapy-induced hand and foot numbness is associated with the loss of IENF. PGP9.5 is a ubiquitin hydrolase widely present in neurons, nerve fibers, and neuroendocrine cells. We used the anti-PGP 9.5 antibodies to label the PGP 9.5-positive fibers and to observe the IENF density in mice hind paw skin tissue.

**Fig. 6:**
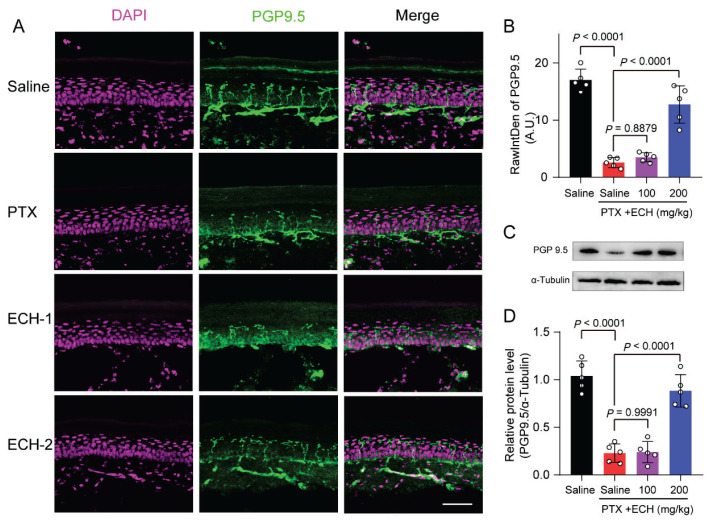
Effect of repeated ECH on the intraepidermal nerve fibers in hind paw skin of mice. (**A**) Sections from hind paws in mice of each group were immune-stained by PGP9.5 (green) and DAPI (red). Scale bar = 100 μm. (**B**) Quantification for PGP9.5 in hind paw skin from mice in each group. (**C–D**) Western blot and quantification of PGP9.5 in mice hind paw skin. one-way ANOVA, followed by Dunnett’s multiple comparisons test. P values are indicated in the Figs. Data are expressed as mean ± SEM. (n = 5 mice/group).

Our results showed that the IENF density was significantly decreased in paclitaxel treated mice hind paw skin (Fig. 6B; *P*<0.0001). Repeated injections of echinacoside 200mg/kg, but not 100mg/kg significantly alleviated this reduction (Fig. 6B; *P*<0.0001, P=0.8879, respectively). We also used Western Blot method to detect the relative expression of PGP9.5 protein, Consistent with the immunofluorescence results, only echinacoside 200mg/kg prophylactic administration significantly prevented the reduction of PGP9.5 protein (Fig. 6C–D; *P*<0.0001).

## Discussion

Echinacoside (ECH), a naturally occurring phenylethanoid glycoside, has demonstrated promising therapeutic potential for neuropsychiatric disorders in preclinical studies [10]. Accumulating evidence indicates that ECH exerts multifaceted neuroprotective effects, including mitochondrial function preservation, oxidative stress reduction, anti-inflammatory activity, endoplasmic reticulum stress attenuation, and autophagy induction [11]. In the present study, we provide novel evidence that ECH (100 mg and 200 mg/kg, intraperitoneally) for 7 days effectively prevents the development of paclitaxel-induced peripheral neuropathy (PIPN) in mice. Our findings demonstrate that repeated ECH administration during PIPN induction significantly alleviated mechanical allodynia and thermal/cold hyperalgesia, suggesting a prophylactic role in neuropathic pain. The beneficial effects of ECH in PIPN were associated with the suppression of spinal microglial and astrocytic activation, along with reduced expression of pro-inflammatory cytokines (IL-1β, TNF-α, IL-6). These findings align with previous reports on ECH’s anti-neuroinflammatory properties [12]. Furthermore, ECH treatment attenuated the loss of plantar nerve fibers, indicating its direct neuroprotective function. Given that neuroinflammation and peripheral nerve degeneration are key contributors to chemotherapy-induced neuropathy (CIPN) [13], our results suggest that ECH may act through multiple mechanisms to counteract PIPN pathogenesis.

Our findings suggest that ECH alleviates chemotherapy-induced neuropathic pain. Notably, existing studies have demonstrated that ECH itself possesses potent antitumor effects. Researches show that ECH could trigger cell cycle arrest and apoptosis in SW480 cancer cells by causing oxidative DNA damage [14]. ECH could inhibit cell proliferation, invasion and migration, and promoted the apoptosis of breast cancer cells by downregulating the expression of miR-4306 and miR-4508 [15]. ECH also could promote the activation of the TGF-β1/Smad signaling pathway and increased the expression levels of Bax/Bcl-2 in liver cancer cells [16]. Therefore, this study provides a theoretical foundation and feasibility for the clinical application of ECH, as well as its potential use in combination with chemotherapeutic agents.

Paclitaxel-induced peripheral neuropathy (PIPN) is closely associated with the aberrant activation of glial cells in both the central and peripheral nervous systems [17]. Studies demonstrate that the activation of microglia and astrocytes exacerbates neuroinflammation by releasing pro-inflammatory cytokines (e.g., TNF-α, IL-1β, IL-6) and neuroactive substances (e.g., ATP, CX3CL1), leading to mechanical allodynia and thermal hyperalgesia [17]. Paclitaxel activates spinal dorsal horn microglia via the TLR4/NF-κB pathway, promoting the release of inflammatory mediators and enhancing neuronal excitability (e.g., through the BDNF-TrkB signaling axis) [18].

Inflammatory factors released by microglia further activate astrocytes, sustaining chronic pain states via JAK2-STAT3 or MAPK signaling pathways [19]. The tripartite signaling network among microglia, astrocytes, and neurons plays a pivotal role in the persistence of paclitaxel-induced neuropathic pain [20]. Our findings suggest that ECH alleviates paclitaxel-induced microglia and astrocytes activation in mice spinal cord horn, According to published studies, ECH could suppress neuroinflammation through the TLR4/MyD88 pathway, ECH is likely to exert its inhibitory effect on microglial activation by modulating the TLR4/MyD88 pathway at the spinal level, which warrants further investigation.

Our study demonstrated that ECH significantly reduced the levels of pro-inflammatory cytokines (IL-1β, TNF-α, and IL-6) in the spinal cord, which aligns with the findings reported by Zhu et al. that ECH suppresses neuroinflammation through the TLR4/MyD88 pathway [21]. Research has demonstrated that ECH regulated the IL-6/JAK2/STAT3 pathway and STAT3 phosphory-lation, leading to neurotrophic and anti-inflammatory effects [22].

Chemotherapeutic agents like paclitaxel generally induce neurotoxicity through disruption of axonal microtubule structure [23] and subsequent impairment of axonal transport, mitochondrial dysfunction in primary afferent neurons, and excessive generation of reactive oxygen species [24]. These pathological changes ultimately lead to damage of nerve terminal fibers responsible for temperature sensation and nociception [25]. Quantification of intraepidermal nerve fiber (IENF) density revealed that ECH significantly attenuated paclitaxel-induced loss of plantar nerve fibers in mice. This finding mechanistically aligns with ECH’s established roles in mitochondrial protection [26]and antioxidative stress [12], suggesting its multimodal action in preserving peripheral nerve structural integrity.

## Conclusions

In conclusion, our study demonstrated a notable analgesia effect of ECH in the PIPN model. This research demonstrates that repetitive administration of echinacoside can alleviate neuropathic pain behaviors and peripheral nerve fiber loss induced by paclitaxel, which may through its inhibitory effect on spinal inflammation. Thus, our results suggest that echinacoside treatment is a promising approach for the management of PIPN.
